# Editorial: Effects of physical activity on psychological well-being

**DOI:** 10.3389/fpsyg.2023.1121976

**Published:** 2023-01-18

**Authors:** Nebojša Trajković, Petar M. Mitić, Renata Barić, Špela Bogataj

**Affiliations:** ^1^Faculty of Sport and Physical Education, University of Niš, Niš, Serbia; ^2^Faculty of Kinesiology, University of Zagreb, Zagreb, Croatia; ^3^Department of Nephrology, University Medical Centre Ljubljana, Ljubljana, Slovenia

**Keywords:** exercise, behavioral changes, health, psychology, children, adults

The effects of regular physical activity on overall health are well documented in the literature. Sabe et al. (2022) conducted a comprehensive scientometric analysis to evaluate topics and trends published in the Web of Science Core Collection between 1905 and 2021. The authors found an exponential annual growth rate of 6.8% since 1989 and identified seven major trends in physical activity research: cardiovascular disease, somatic disorders, cognitive decline/dementia, mental illness, sports performance, health and eating disorders, and the pandemic COVID-19. The preventive role of a physically active lifestyle is well established, and several strategies aim to get and keep the general population active to achieve better physical and mental health and a higher quality of life. In addition, the role of physical activity in public health is emphasized, and it is important to involve kinesiologists and sports professionals as stakeholders in the decision-making process, which affects the general population. Non-adherence to physical activity recommendations is an important risk factor for several somatic diseases as well as mental health problems, mental illness (Firth et al., 2020), and brain and cognitive function (Erickson et al., 2019). Regular exercise can improve social relationships, identity, sense of belonging (Stevens et al., 2021), self-confidence, self-efficacy, and body image (Kouhi et al., 2011; Abarca-Sos et al., 2015; Hao et al., 2022), which are psychological predictors of overall wellbeing. Psychological wellbeing is associated with a lower risk of disease and mortality and can be improved through relatively low-cost interventions (Boehm et al., 2012; Trudel-Fitzgerald et al., 2019). Factors known to influence mental and physical health include age, gender, chronic disease, amount and type of physical activity, environmental factors, and adaptation to emotional and physical stressors (Meyer et al., 2014; Gallagher et al., 2015).

Physical activity causes certain physiological and biochemical changes in the brain and body, as well as some changes in the perception and experience of the environment and one's own body, which contribute to improved psychological functioning. Despite these findings, the psychological benefits of regular physical activity and the psychological predictors that may influence regular physical activity are less clear. As a result, studies examining the relationship between physical activity and psychological health have become increasingly important in recent years (Ruegsegger and Booth, 2018; Kandola et al., 2019).

In this Research Topic, we address the effects of physical activity and exercise interventions on general and mental health and disease-related outcomes. In addition, we examine the relationships between physical activity/functional skills and mortality, cognition, and disease burden. This Research Topic forms a multidisciplinary perspective, covering the fields of kinesiology, medicine, psychology, and physical therapy. The articles in the Research Topic present a multidisciplinary approach to the relationships between different forms of physical activity (type, intensity, frequency, and competitive/recreational) and psychological wellbeing, and aim to provide answers on how to maximize the potential positive effects of physical activity on psychological functioning in people with different characteristics.

Physical activity, regular sleep, fasting, and autophagy can be good ways to achieve wellbeing and longevity. However, to achieve healthy longevity and wellbeing, a combination of all four methods is required, rather than using only one (Min et al.). In addition, the aging process leads to various changes [functional abilities, fitness level, physical activity, and body mass index (BMI)], which in turn lead to changes in quality of life. It has been proven that, in the domain of physical activity, there is a correlation with the quality of life of older women (Ðošić et al.). A research protocol on cognitive training combined with physical exercise in relation to cognitive functions, physical performance, and indicators of frailty in patients with hemodialysis has been discussed in detail by Bogataj et al..

Mladenović et al. noted that the COVID-19 pandemic has certain effects on people's mental health, and these effects have not yet been fully studied. When completing the Serbian version of the Multidimensional Emotion Questionnaire (MEQ), negative differences were found in terms of engagement by gender and sport, but not for the scale of regulation of positive emotions. It has also been shown that participation in sports can contribute to the improvement of emotional state, especially in situations such as during the COVID-19 pandemic. Another study (Živković et al.) found possible changes in life satisfaction of students participating in recreational sports, explained by the pandemic. Extraversion had significant predictive power for subjective experience of life satisfaction, whereas neuroticism and psychoticism did not. However, Marinkovic et al. showed that a neuromuscular exercise program can significantly improve the quality of life of young and healthy people following COVID-19 infection.

Several studies included the younger population and studied how exercise and physical activity affect their mental health. Carton et al. found that older adolescents derived greater positive affective benefits from membership in sports clubs compared to younger adolescents. Older adolescents may also use the affective benefits of sports club membership to gain advantages for the first steps in their adult lives, such as attending university or entering the workforce. Physical activity is one of the most important ways to maintain health, while life satisfaction is an indicator of students' mental health. After completing questionnaires (Exercise Adherence Questionnaire, Core Self-evaluation Scale, Positive Affect and Negative Affect Scale, and Satisfaction with Life Scale), it was found that there was a strong relationship between physical activity and life satisfaction among university students. In addition, relationships were found between physical activity, basic self-evaluation, positive emotions, and life satisfaction (Liu F. et al.). Šalaj and Masnjak found a weak relationship between children's motor skills and their social-emotional skills. Preschool children with high or low motor dispositions did not differ in terms of the occurrence of social and emotional difficulties. On the contrary, physical activity was found to have a large impact on final math grades. For boys, muscle endurance, aerobic capacity, and upper body strength were the most common predictors of final math grade, while for girls it was coordination (Sember et al.). Zovko et al. showed that mothers' moderate-to-vigorous physical activity was related to children's moderate-to-vigorous physical activity, whereas fathers' time spent in sedentary activities was related to boys' sedentary behavior but not to girls'. Grandparents' physical activity was not a significant predictor of children's physical activity, whereas grandfathers' sedentary behavior was a significant predictor of children's sedentary behavior. Zhang et al. found that physical literacy (PL) is an integral part of physical education and physical activity. The authors' study found that there was a positive relationship between cardiovascular fitness and PL. Self-confidence and physical competence have been shown to be more positively related to aspects of physical fitness (PF) in Chinese university students. Therefore, the use of PL has been shown to be a good tool to improve PF.

Unfortunately, mental health is becoming an increasing problem in the younger population, especially post-pandemic. Yu et al. found that students with myopia or other forms of impairment were more likely to suffer from anxiety. Myopia, irrational eating habits, and injuries related to physical activities were also factors leading to anxiety and stress. Risk factors for depression included low parental education, irrational eating habits, male gender, and low levels of physical activity.

Two studies dealt with professional athletes. Stanković et al. found that professional judoka were characterized by low levels of aggression, especially low expression of indirect and physical aggression. The personality traits honesty and openness to experience were also high, while emotionality and extraversion were lower. Moderate general self-efficacy was also a characteristic of professional judoka. Members of team sports, on the other hand, were characterized by increased aggression, pronounced emotionality and extraversion traits, and less pronounced honesty and openness to new experiences traits as well as general self-efficacy. Another study on athletes (Copec et al.) supports the current literature and suggests that the construct of stress attitude plays a unique role in explaining individual differences in cognitive stress ratings across general personality dimensions.

Liu X. et al. concluded that Tai Chi and Qigong exercises have a positive effect on reducing depression and anxiety and lowering cortisol levels in adolescents. However, the effects on mood, stress, and self-esteem were found to be very small. Therefore, it may be stated that Qigong is a good therapeutic tool for improving the psychological state of adolescents. Similarly, Yang et al. found in their systematic review that traditional Chinese fitness exercises can improve both negative moods and sleep disorders. On the contrary, Hammer et al. found that negative consequences occur with high-intensity interval training. However, these effects can be reduced if the intensity is maintained at approximately 80% maximum heart rate, with positive enjoyment reactions associated with interval training.

## Conclusion

The current Research Topic contributes to the existing literature by providing information on the relationships between physical activity and psychological function and wellbeing. Studies have shown that physical activity contributes to a healthy life expectancy and quality of life, as well as cognitive function in various populations. The positive psychological effects of physical activity and exercise include increased life satisfaction, positive emotions, self-appraisal, self-efficacy, self-confidence, and physical competence, as well as improved socio-emotional skills in children. Higher levels of physical activity are also associated with lower cortisol levels, lower negative mood, fewer symptoms of depression and anxiety, and fewer sleep disturbances; physical activity may be considered a protective factor for lower stress levels and stress attitudes, depression, poor eating habits, and exercise habits. In conclusion, it was found that there are some additional positive predictors of these positive effects related to physical activity, such as personality traits (extraversion, openness), parents' physical activity level, moderate intensity of physical activity, and level of physical education. However, it is important to point out that this is not only due to participation in sports or physical activity itself, but also to how these are conducted and organized.

The work published in this Research Topic raises important questions for future investigations, including (1) the role of the sport environment as a mediator of these positive effects, (2) the potential differential effects of recreational and competitive physical activity on psychological wellbeing, (3) additional investigation of the therapeutic role of physical activity participation in different patient populations or in individuals with specific impairments in terms of psychological wellbeing and quality of life, and (4) investigation of the differential effects of physical activity on wellbeing in different age groups.

## Author contributions

All authors listed have made a substantial, direct, and intellectual contribution to the work and approved it for publication.
